# FungiQuant: A broad-coverage fungal quantitative real-time PCR assay

**DOI:** 10.1186/1471-2180-12-255

**Published:** 2012-11-08

**Authors:** Cindy M Liu, Sergey Kachur, Michael G Dwan, Alison G Abraham, Maliha Aziz, Po-Ren Hsueh, Yu-Tsung Huang, Joseph D Busch, Louis J Lamit, Catherine A Gehring, Paul Keim, Lance B Price

**Affiliations:** 1 Division of Pathogen Genomics, Translational Genomics Research Institute, Flagstaff, AZ, 86011, USA; 2 Center for Microbial Genetics and Genomics, Northern Arizona University, Flagstaff, AZ, 86011, USA; 3 Department of Epidemiology, The Johns Hopkins Bloomberg School of Public Health, Baltimore, MD, 21201, USA; 4 Departments of Laboratory Medicine and Internal Medicine, National Taiwan University Hospital, National Taiwan University College of Medicine, Taipei, Taiwan; 5 Department of Internal Medicine, Far Eastern Memorial Hospital, New Taipei City, Taiwan; 6 Department of Biological Sciences, Northern Arizona University, Flagstaff, AZ, 86011, USA; 7 Current address: Ross University School of Medicine, Roseau, Dominica

## Abstract

**Background:**

Fungal load quantification is a critical component of fungal community analyses. Limitation of current approaches for quantifying the fungal component in the human microbiome suggests the need for new broad-coverage techniques.

**Methods:**

We analyzed 2,085 18S rRNA gene sequences from the SILVA database for assay design. We generated and quantified plasmid standards using a qPCR-based approach. We evaluated assay coverage against 4,968 sequences and performed assay validation following the Minimum Information for Publication of Quantitative Real-Time PCR Experiments (MIQE) guidelines.

**Results:**

We designed FungiQuant, a TaqMan^®^ qPCR assay targeting a 351 bp region in the fungal 18S rRNA gene. Our *in silico* analysis showed that FungiQuant is a perfect sequence match to 90.0% of the 2,617 fungal species analyzed. We showed that FungiQuant’s is 100% sensitive and its amplification efficiencies ranged from 76.3% to 114.5%, with *r*^*2*^-values of >0.99 against the 69 fungal species tested. Additionally, FungiQuant inter- and intra-run coefficients of variance ranged from <10% and <20%, respectively. We further showed that FungiQuant has a limit of quantification 25 copies and a limit of detection at 5 copies. Lastly, by comparing results from human-only background DNA with low-level fungal DNA, we showed that amplification in two or three of a FungiQuant performed in triplicate is statistically significant for true positive fungal detection.

**Conclusions:**

FungiQuant has comprehensive coverage against diverse fungi and is a robust quantification and detection tool for delineating between true fungal detection and non-target human DNA.

## Background

Fungi are among the most diverse eukaryotic organisms on Earth, with nearly 10,000 named fungal species and an estimated 1.5 to 5 million species that are yet to be defined [1,2]. Fungi are also recognized as an important element in human microbiome research, clinical medicine, and as emerging pathogens [3-8]. However, methodological challenges have limited scientists’ and clinicians’ ability to detect and measure fungal abundance.

Currently, fungal detection is performed through culturing [9], serological detection of antigens, such galactomannan in invasive aspergillosis [10,11], and molecular test panels [12]. Yet, these methods lack broad-coverage and are not quantitative [4,13]. Next-generation sequencing is an effective approach for detecting and characterizing fungi, but it is expensive, requires complex analyses, and is not quantitative [14,15].

Measurements of fungal abundance are now typically performed using biochemical methods targeting ergosterol, chitin, and fatty acid profiles [16-18], which often require extraction methods that exclude further molecular analyses and can lack coverage against major fungal lineages [19]. Alternative approaches such as microscopy [20] and quantitative culture [21,22] are also time-consuming, operator-dependent, and lack broad-coverage.

To address these limitations, a quantitative molecular tool that is broad-coverage, sensitive, and specific is needed [23,24]. Together with qualitative characterization of fungi, such a tool will provide a comprehensive view of the fungal microbiota. Additionally, this broad-coverage fungal quantification tool can be used independently to measure fungal abundance changes over time, in response to treatment, or among multiple study groups.

Quantitative real-time PCR (qPCR) has been shown to be more sensitive than culture-based approaches against a wide range of fungal species [25]. Much progress has been made in developing qPCR assays that can detect diverse fungal species [26-30], but we sought to develop a qPCR assay that would approach universal fungal coverage. In the current manuscript, we present our design of a broad-coverage qPCR assay—FungiQuant—for fungal detection and quantification targeting the fungal 18S rRNA gene. We performed both *in silico* analysis based on primer and probe sequence matches to reference fungal 18S rRNA gene sequences and laboratory validation following the Minimum Information for Publication of Quantitative Real-Time PCR Experiments (MIQE) guidelines [31]. Lastly, we established guidelines for quantification and detection analysis based results from triplicate reactions using FungiQuant.

## Methods

### Design of fungal 18S rRNA gene quantitative real-time PCR (qPCR) assay

We downloaded fungal 18S rRNA gene sequences alignment scores and sequence quality scores of >90 and have a length of 1400 bp or longer from SILVA Release 93 (n = 2,085) [32]. We summarized the aligned sequences the occurrence of each allele at each nucleotide position. Alignment positions with a gap content of >97% were excluded.

We identified a highly conserved 500 bp region for qPCR assay design. In our assay design, we stipulated that: 1) primers can only have three or fewer degenerate bases and 2) the probe contains no degenerate bases. Using the allele occurrence analysis file, we incorporated key degenerate bases into each primer and designed a non-degenerate probe. The primer T_m_ was calculated using OligoCalc [33] and the probe T_m_ was calculated using the Primer Probe Test Tool from the Primer Express^®^ Software for Real-Time PCR version 3.0 (Applied Biosystems by Life Technologies, Carlsbad, CA, USA) (Table 1).

**Table 1:** FungiQuant primer and probe sequences

**FungiQuant (351 bp)**		**Tm *(°C)***	***S. cerevisiae *region**
FungiQuant-F	5^′^-GG**R**AAACTCACCAGGTCCAG-3^′^	60.5-62.5	*1199-1218*
FungiQuant-R	5^′^-G**SW**CTATCCCCA**K**CACGA-3^′^	56.3-58.4	*1269-1283*
FungiQuant-Prb	(6FAM) 5^′^-TGGTGCATGGCCGTT-3^′^ (MGBNFQ)	68.0	*1532-1549*

### Computational analysis of assay specificity and coverage

A **Specificity analysis.** We assessed assay specificity using megablast against human and bacterial sequences from the Genbank nucleotide collection (nr/nt) [34].

B **Collection of 18S rRNA gene sequence for*in silico*coverage analysis.** From SILVA Release 108, we downloaded the sequences, sequence ID, and Genbank accession numbers of all fungal 18S rRNA gene sequences with sequence quality score of >90 and are 1,400 bp or longer [32]. We extracted the full Genbank taxonomy for each sequence, which we concatenated (e.g., at order-level, a taxonomic identification consists of phylum-subphylum-class-order). We replaced empty data fields in the concatenated taxonomy with “unknown”, when applicable.

C **Overview of*in silico*assay coverage analysis.** We performed the *in silico* coverage analysis using a stringent and a relaxed criterion, where the stringent criterion requires full perfect match of both primers and the relaxed criterion requires perfect match of the last eight nucleotides at the 3’ end of the primers. Both conditions require full perfect match of the probe sequence. For each condition, we determined the assay’s numerical and taxonomic coverage at the phylum, sub-phylum, class, order, family, genus, and species levels. Details for the *in silico* coverage analysis can be found in the Additional file 1: Methodological Details.

### Quantification and normalization of FungiQuant plasmid standards

We utilized a qPCR-based approach to quantify and normalize the FungiQuant plasmid standards, a *C. albicans* 18S rRNA gene clone, to a Cp-value equivalent to 10^9^ copies/μl. Details for FungiQuant plasmid normalization can be found in the Additional file 1: Methodological Details.

### FungiQuant optimization and specificity check

After testing multiple primer and probe concentrations, the optimized conditions included 10 μl and 5 μl of reaction volumes using 1 μl of template, with the final reaction containing 1.8 μM of each forward and reverse primer, 225 nM the TaqMan^®^ probe, 1% formamide, 1X Platinum^®^ Quantitative PCR SuperMix-UDG w⁄ROX (Invitrogen Corp.) and molecular-grade water. We included an in-run standard curve (25 copies, 50 copies, and 10^2^-10^7^ copies in 10-fold serial dilutions) and no-template controls in each run, with all reactions performed in triplicates on the 7900HT Real Time PCR System (Applied Biosystems). We used the following PCR conditions: 3 min at 50°C for UNG treatment, 10 min at 95°C for *Taq* activation, 15 s at 95°C for denaturation and 1 min at 65°C for annealing and extension x 50 cycles. We determined the Ct-value for each reaction using a manual Ct threshold of 0.10 and automatic baseline in the Sequence Detection Systems v2.3 software (Applied Biosystems). Using the optimized assay condition, we tested FungiQuant against 0.5 ng, 1 ng, 5 ng, and 10 ng of human genomic DNA (Promega, Madison, WI, USA) mixed with the normalized plasmid standards in triplicate reactions.

### FungiQuant laboratory evaluation using diverse fungal genomic DNA

To assess FungiQuant’s performance against diverse fungi, we evaluated the assay efficiency and correlation coefficients against a collection of fungal genomic DNA, details regarding the fungal DNA collection can be found in Additional file 1: Methodological Details.

### Experimental design

For sensitivity and efficiency analysis, we tested each fungal genomic DNA in three 10-fold serial dilutions in triplicate reactions using the optimized 18S qPCR conditions as described above. Using the Ct-value results, we calculated FungiQuant’s reaction efficiency and correlation coefficient for each species tested.

### Limit of detection (LOD) validation

#### Experimental design

To determine the LOD of FungiQuant for detecting low concentration fungal DNA, we analyzed no-template controls (i.e., molecular grade H2O), background control (i.e., 10 ng, 50ng, and 150ng human DNA), as well as three low concentration of fungal DNA: a) 1.8 copies, b) 5 copies, and c) 10 copies of fungal 18S rRNA gene. Each template was analyzed in 96 replicates in 10 μl and 5 μl reactions using conditions as described above.

#### Data Analysis

Experimental results using all templates were assessed for: a) the proportion of determined and undetermined values and b) the Ct-value distribution among those replicates with determined values. Using the specificity associated with the background controls, which provides the most likely source of contamination and signal noise, the probability of each triplicate results was calculated under the null hypothesis that the sample contained no positive target. The analysis was performed separately for each reaction volume using an alpha level of 0.05 to determine results inconsistent with the null. Analysis using the Ct-value from samples with positive amplification was also performed using a non-parametric median test to determine if 1.8 copies, 5 copies, or 10 copies templates could be differentiated from the no-template and background controls. The Ct-value data was further assessed to determine if the average Ct-value is an appropriate estimate of the true Ct-value in low concentration samples for reporting and analysis.

#### FungiQuant laboratory quantitative validation

##### Experimental design

We followed the Minimum Information for publication of Quantitative real-time PCR Experiments, or the MIQE guidelines, whenever applicable [31]. We performed additional tests to evaluate FungiQuant performance when background human DNA is present. We included seven template conditions: plasmid standards alone and plasmid standards with 0.5 ng, 1 ng, 5 ng, and 10 ng of human DNA per reaction in 10 μl reactions, as well as plasmid standards alone and plasmid standards with 1 ng human DNA in 5 μl reactions. For each condition assessed, we performed three qPCR runs to assess reproducibility. In each run, three replicate standard curves were tested across the 384-well plate to assess repeatability. Details for the data analysis can be found in Additional file 1: Methodological Details.

##### Fungi-to-human DNA threshold ratio calculations

We determined FungiQuant’s minimum threshold of fungi-to-human DNA ratio using an estimate of average human 18S rRNA gene copy number per genome as 400 copies [35]. We estimated the diploid human genome as 5,758 Mb [36] or the mass equivalent of 5,758Mb/(0.978x10^3^ Mb/pg) = 5.887 pg per diploid human genome [37].

## Results

### FungiQuant assay design

We identified three highly conserved regions based on analysis results of a high-quality 18S rRNA gene multiple sequence alignments. Within these conserved regions, we designed two degenerate primers and a non-degenerate TaqMan^®^ minor-groove binding probe (Table 1). We positioned the probe on the reverse strand, proximal to the forward primer to create favorable thermodynamic profile and maximize assay specificity (Additional file 1: Table S1).

### *in silico* analysis of FungiQuant assay coverage using 18S rRNA gene sequences from 18 fungal subphyla

We performed *in silico* coverage analysis using a stringent and a relaxed criterion against 4,968 18S rRNA gene sequences, encompassing 18 fungal subphyla. Based on the stringent criterion, we showed that 15 of the 18 subphyla had perfect sequence matches to FungiQuant (Table 2). We found that most covered subphyla were substantially covered on the genus-level as well, typically with 90% or more of the genera being perfect sequence matches. Exceptions included Mucoromycotina (20/36; 55.56%), Kickxellomycotina (6/9; 66.67%), and Chytridiomycota (9/13; 69.23%). Microspordia and Entomophthoromycotina were the two subphyla without any perfect sequence matches to FungiQuant (Additional file 2: Figure S1). We found that 1,018 genera (91.4%) and 2,355 species (90.0%) had at least one perfect sequence match to FungiQuant (Table 2).

**Table 2:** **Results from the *in silico *coverage analysis performed using two sequence matching conditions**

	**Full length primer & probe (Stringent)**	**8-nt primer & full length probe (Relaxed)**
Phylum	**77.8%**	**88.9%**
	(7/9)	(8/9)
Sub-phylum	**83.3%**	**94.4%**
	(15/18)	(17/18)
Class	**92.3%**	**97.4%**
	(36/39)	(38/39)
Order	**91.3%**	**96.9%**
	(116/127)	(123/127)
Family	**91.9%**	**95.4%**
	(342/372)	(354/372)
Genus	**91.4%**	**94.9%**
	(1018/1114)	(1057/1114)
Species	**90.0%**	**94.2%**
	(2355/2617)	(2465/2617)

**Figure 1:**
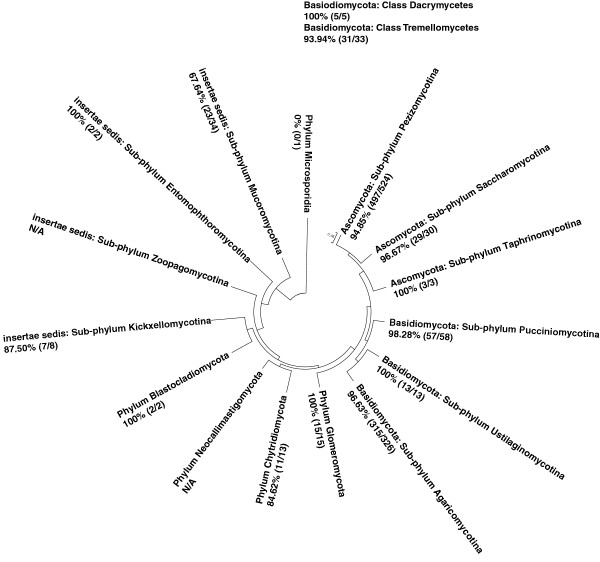
**FungiQuant *in silico *coverage analysis using the relaxed criterion against 993 genera and 9 phyla, demonstrating broad-coverage.** On the 18S rRNA gene-based phylogeny, each analyzed fungal phylum is annotated with its genus-level FungiQuant coverage based on the relaxed criterion. This is presented as a numerator (i.e., the number of covered genus for the phylum), a denominator (i.e., the number of genera eligible for sequence matching for the phylum), and the percentage of coverage.

When we applied the relaxed criterion, we determined that FungiQuant covered Entomophthoromycotina (Figure 1). We also found that 1,057 genera (94.9%) and 2,465 species (94.2%) had at least one perfect sequence match to FungiQuant (Table 2). In addition, we determined that FungiQuant had excellent coverage for many clinically relevant genera such as *Cryptococcus spp.* (49/49; 100%), *Fusarium spp.* (7/7; 100%), *Mucor spp.* (7/7; 100%), *Rhizopus spp.* (15/15; 100%), and *Candida spp.* (108/119; 90.76%). Analysis also showed comprehensive coverage for common environmental genera such as *Glomus spp.* (24/25; 96.00%), *Gigaspora spp.* (5/5; 100%), *Trichosporon spp.* (31/31; 100%), and *Rhodotorula spp.* (22/22; 100%). Detailed results for the coverage analysis can be found in Additional file 3: Table S4, Additional file 4: Table S5.

### FungiQuant sensitivity against diverse fungal DNA

We tested the sensitivity of FungiQuant against 69 clinical and environmental species from seven subphyla in the laboratory. We showed that FungiQuant is 100% sensitive against these diverse species from Agaricomycotina (n = 22), Mucormycotina (n = 4), Pezizomycotina (n = 29), Pucciniomycotina (n=2), Saccharomycotina (n = 17), Taphrinomycotina (n = 1), and Ustilaginomycotina (n = 1) (Table 3). All of the fungal species tested were perfect sequence matches to FungiQuant, and based on results from three ten-fold dilutions, we found that the assay reaction efficiencies ranged from 76.29% to 114.45%., with *r*^*2*^-value of >0.99 (Table 3).

**Table 3:** FungiQuant sensitivity and reaction efficiency against diverse fungal species

**Subphylum**	**Species**	**Reaction efficiency**	***r***^***2***^
Saccharomycotina	*Debaryomyces hansenii*	101.42%	*>0.99*
Saccharomycotina	*Lodderomyces elongisporus*	93.04%	*>0.99*
Taphrinomycotina	*Schizosaccharomyces pombe*	97.38%	*>0.99*
Saccharomycotina	*Candida albicans*	89.95%	*>0.99*
Pezizomycotina	*Acremonium strictum*	78.95%	*>0.99*
Pezizomycotina	*Aspergillus flavus*	85.96%	*>0.99*
Pezizomycotina	*Aspergillus fumigatus*	81.85%	*>0.99*
Pezizomycotina	*Aspergillus niger*	113.61%	*>0.99*
Pezizomycotina	*Aspergillus versicolor*	89.59%	*>0.99*
Pezizomycotina	*Aureobasidium pullulans*	84.08%	*>0.98*
Pezizomycotina	*Chaetomium globosum*	85.44%	*>0.99*
Pezizomycotina	*Elaphomyces decipiens*	94.78%	*>0.99*
Pezizomycotina	*Exophiala dermatitidis*	76.29%	*>0.99*
Pezizomycotina	*Fusarium equiseti*	89.66%	*>0.99*
Pezizomycotina	*Fusarium oxysporum*	99.70%	*>0.98*
Pezizomycotina	*Fusarium solani*	103.38%	*>0.99*
Pezizomycotina	*Microsporum canis*	84.23%	*>0.99*
Pezizomycotina	*Neurospora crassa*	90.65%	*>0.99*
Pezizomycotina	*Paecilomyces lilacinus*	90.69%	*>0.99*
Pezizomycotina	*Paecilomyces sinensis*	82.30%	*>0.99*
Pezizomycotina	*Paecilomyces variotii*	95.15%	*>0.99*
Pezizomycotina	*Penicillium marneffei*	96.54%	*>0.99*
Pezizomycotina	*Scedosporium apiospermum*	91.58%	*>0.99*
Pezizomycotina	*Sporothrix schenckii*	90.86%	*>0.99*
Pezizomycotina	*Trichophyton mentagrophytes*	92.82%	*>0.99*
Pezizomycotina	*Trichophyton rubrum*	91.43%	*>0.99*
Saccharomycotina	*Candida famata*	90.13%	*>0.99*
Saccharomycotina	*Candida guilliermondii*	82.24%	*>0.99*
Saccharomycotina	*Candida haemulonii*	99.82%	*>0.99*
Saccharomycotina	*Candida intermedia*	81.72%	*>0.99*
Saccharomycotina	*Candida quercitrusa*	98.16%	*>0.99*
Saccharomycotina	*Candida tropicalis*	88.28%	*>0.99*
Saccharomycotina	*Geotrichum candidum*	79.76%	*>0.99*
Saccharomycotina	*Pichia ohmeri*	102.31%	*>0.99*
Saccharomycotina	*Saccharomycopsis crataegensis*	80.98%	*>0.99*
Saccharomycotina	*Stephanoascus ciferrii*	85.84%	*>0.99*
Mucoromycotina	*Absidia corymbifera*	92.33%	*>0.99*
Mucoromycotina	*Cunninghamella bertholletiae*	80.03%	*>0.99*
Mucoromycotina	*Rhizopus microsporus*	89.16%	*>0.99*
Mucoromycotina	*Rhizopus oryzae*	87.96%	*>0.99*
Pezizomycotina	*Alternaria* sp.	103.70%	*>0.99*
Pezizomycotina	*Cladosporium cladosporioides*	92.87%	*>0.99*
Pezizomycotina	*Cytospora chrysosperma*	100.50%	*>0.99*
Pezizomycotina	*Endoconidioma* sp*.*	89.93%	*>0.99*
Pezizomycotina	*Geopora* sp.	114.45%	*>0.99*
Pezizomycotina	*Phoma herbarum*	91.94%	*>0.99*
Pezizomycotina	*Xanthomendoza galericulata*	94.27%	*>0.99*
Agaricomycotina	*Agaricus* sp*.*	95.31%	*>0.99*
Agaricomycotina	*Clavulina coralloides*	99.59%	*>0.99*
Agaricomycotina	*Coprinus* sp.	99.70%	*>0.99*
Agaricomycotina	*Cortinarius* sp*.*	102.68%	*>0.99*
Agaricomycotina	*Hebeloma crustuliniforme group*	91.06%	*>0.99*
Agaricomycotina	*Melanogaster* sp*.*	102.27%	*>0.99*
Agaricomycotina	*Pleurotus ostreatus*	102.71%	*>0.99*
Agaricomycotina	*Rhizopogon* sp*.*	107.04%	*>0.99*
Agaricomycotina	*Sclerogaster xerophilus*	92.17%	*>0.99*
Agaricomycotina	*Sedecula pulvinata*	92.26%	*>0.99*
Agaricomycotina	*Tricholoma populinum*	89.53%	*>0.99*
Agaricomycotina	*Trichosporon asahii*	78.03%	*>0.99*
Agaricomycotina	*Trichosporon asteroides*	82.66%	*>0.99*
Agaricomycotina	*Trichosporon cutaneum*	86.66%	*>0.99*
Agaricomycotina	*Trichosporon dermatis*	80.27%	*>0.99*
Agaricomycotina	*Trichosporon faecale*	84.05%	*>0.99*
Agaricomycotina	*Trichosporon montevideense*	77.43%	*>0.99*
Agaricomycotina	*Trichosporon mucoides*	82.87%	*>0.99*
Agaricomycotina	*Trichosporon ovoides*	105.59%	*>0.99*
Pucciniomycotina	*Rhodotorula mucilaginosa*	96.29%	*>0.99*
Pucciniomycotina	*Rhodotorula slooffiae*	99.94%	*>0.99*
Agaricomycotina	*Lactarius* sp*.*	86.76-89.03%	*>0.99*

**Table 4:** FungiQuant quantitative validation results, obtained using pure plasmid standards and different mixed templates

**Templates tested**	**Assay quantitative dynamic range**	**Average reaction efficiency (SD)**	***r***^***2***^**-value**
10 μl Reaction			
Plasmid standards-only	25 – 10^7^ copies	91.80% (1.91%)	*>0.99*
Plasmid standards *plus* 0.5 ng human DNA	25 – 10^7^ copies	93.20% (0.70%)	*>0.99*
Plasmid standards *plus* 1 ng human DNA	25 – 10^7^ copies	97.02% (4.97%)	*>0.99*
Plasmid standards *plus* 5 ng human DNA	25 – 10^7^ copies	92.85% (1.33%)	*>0.99*
Plasmid standards *plus* 10 ng human DNA	25 – 10^7^ copies	91.21% (1.79%)	*>0.99*
*C. albicans* DNA-only	10 fg – 10 ng	94.75% (2.33%)	*>0.98*
*C. albicans* DNA *plus* 1 ng human DNA	10 fg – 10 ng	96.84% (1.93%)	*>0.99*
5 μl Reaction			
Plasmid standards-only	25 – 10^7^ copies	92.17% (5.64%)	*>0.98*
Plasmid standards *plus* 1 ng human DNA	25 – 10^7^ copies	94.21% (2.92%)	*>0.99*
Plasmid standards *plus* 10 ng human DNA	50 – 10^8^ copies	92.64% (2.39%)	*>0.99*

**Table 5:** **Interpretation of FungiQuant results for detecting fungal DNA (i.e., rejecting the Null Hypothesis) based on triplicate 5** μ**l and 10** μ**l reactions**

**Triplicate pattern**	**Probability under the Null Hypothesis (*Ho*: human DNA only)**
10 μl Reaction	
**+++**	**<0.001**
**++−**	**0.008**
+−−	0.077
---	0.744
5 μl Reaction	
**+++**	**0.006**
**++−**	**0.026**
+−−	0.120
---	0.557

### FungiQuant amplification and quantitative profiles against pure plasmids, *C. albicans* DNA, and templates with background human DNA

**Figure 2:**
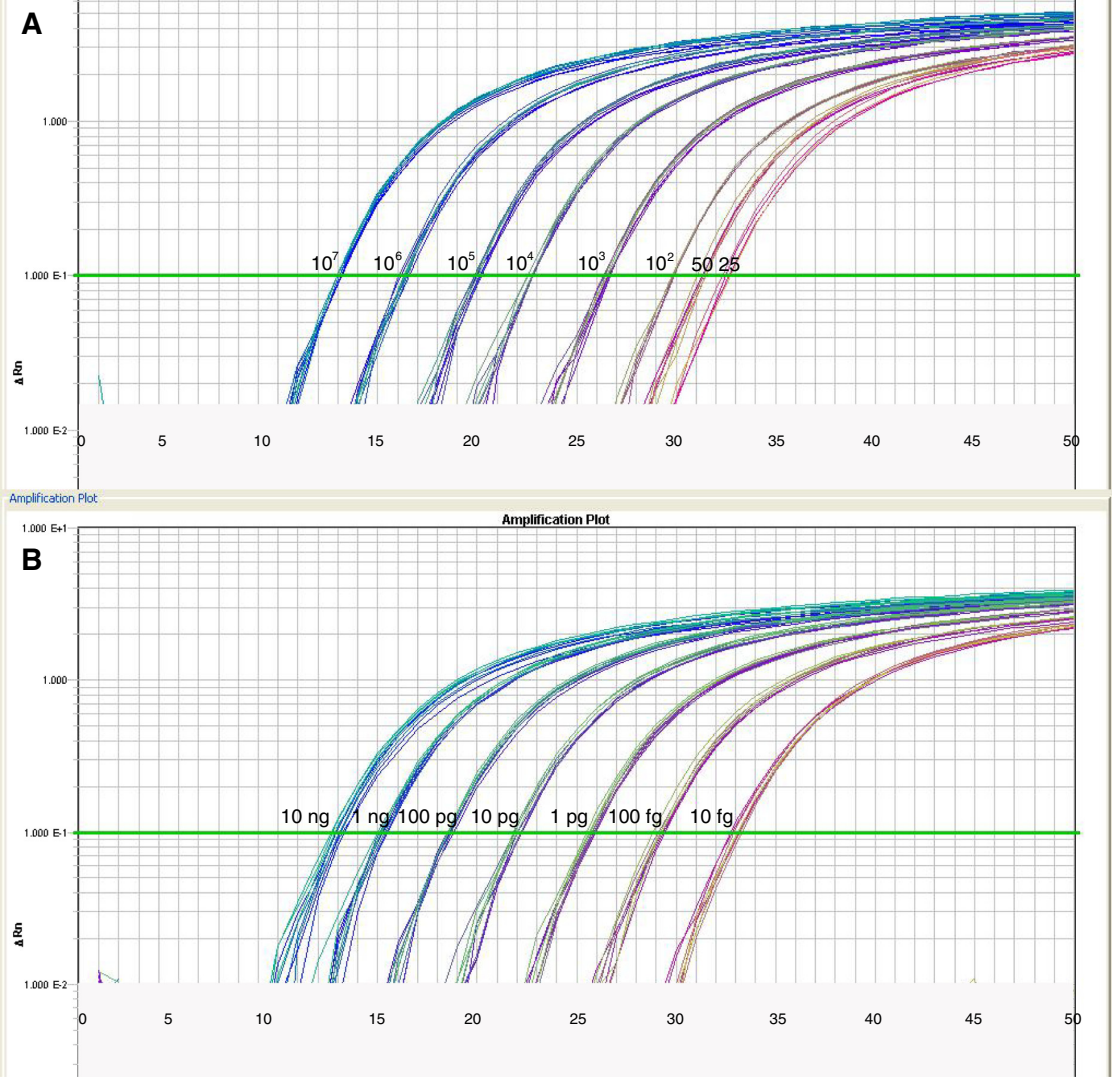
**A-B. FungiQuant amplification profiles.** The FungiQuant amplification profiles remain consistent, irrespective of reaction volume and type of DNA template. The amplification profiles of plasmid standards (Fig. 2**A**) and *C. albicans* DNA (Fig. 2**B**) in two reaction volumes (5 μl and 10 μl) are presented.

We showed FungiQuant had excellent amplification profiles against *C. albicans* plasmid standards and *C. albicans* DNA, with quantitative dynamic range of 25 – 10^7^ copies and 10 fg – 10 ng *C. albicans* DNA, respectively (Figure 2A-B). A list of fungal species that are perfect matches to *C. albicans* in the FungiQuant primer and probe region can be found in Additional file 5: Table S6.

**Figure 3:**
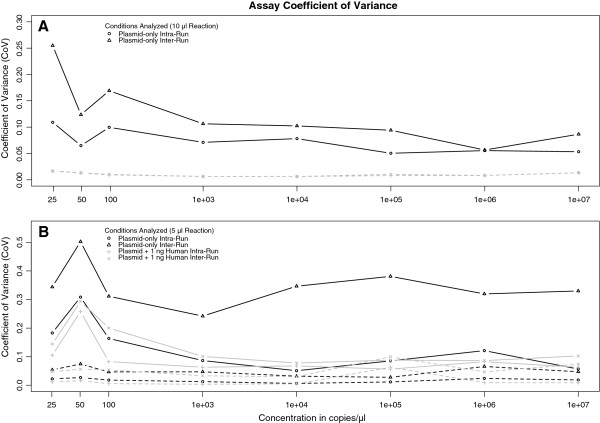
**A-B. FungiQuant inter- and intra-run coefficient of variation (CoV).** FungiQuant CoV is presented for copy number (*solid line*) and Ct-value (*dashed line*), demonstrating the range of CoV, which is lower for the 10 μl than the 5 μl reactions. For the 10 μl reactions, the FungiQuant intra-run copy number CoV is consistently below 15% until at 25 copies, and for the 5 μl reactions, the intra-run CoV is below 20% until at 50 copies. The FungiQuant Ct-value CoV is consistently below 10%, irrespective of reaction volumes.

We also showed that FungiQuant had strong reproducibility, even when we added background human DNA. The inter-run coefficients of variance (CoV) ranged from 0.37 – 3.80% and 3.52 – 34.39% for Ct-value and copy number, respectively. The intra-run average CoV were 0.35 – 2.90% and 1.98 – 23.74% Ct-value and copy number, respectively (Figure 3, Additional file 6: Figure S2). We found that 5 μl reactions had greater inter-run CoV than 10 μl reactions (Figure 3). This suggests that the 10 μl reaction volumes is better suited for quantitative use.

We further determined that FungiQuant’s amplification profile and assay dynamic range were not impacted by the presence of human DNA, at up to 10 ng (Table 4, Additional file 7: Figure S3A-D). Thus, FungiQuant is robust quantitatively even when the fungal 18S rRNA gene is relatively rare as compared to background human DNA. Specifically, we showed that FungiQuant could be applied quantitatively at a ratio of 25:679,464 fungal-to-human 18S rRNA gene copy number.

### FungiQuant is robust for low number of fungal 18S rRNA gene

To validate FungiQuant use for samples with low fungal DNA and high human DNA, we developed guidelines for interpreting triplicate reactions. Additional file 1: Table S2 provides the sensitivity and specificity results from FungiQuant evaluation against multiple positive and negative controls in 10 μl and 5μl reaction volumes. Our analysis showed that FungiQuant could consistently detect 5 copies of 18S rRNA gene template, whereas 1.8 copies were less consistently detected. Nevertheless, further analysis showed that two or more amplification in triplicate reactions is a reliable indicator of positive fungal DNA detection, irrespective of Ct-value(s) obtained (Table 5). These results held for both of the reaction volumes tested.

We also calculated the false negative rate for FungiQuant using the sensitivity associated with 1.8 copies of positive target, a template concentration that provided relatively poor determination. Using a threshold of ≤ 1 positive amplification used for rejecting triplicate results as noise, we determined that the false negative rate could be as high as 80% for samples containing ≤ 1.8 copies when 10 μl reactions are used, and even higher at 87% for samples analyzed using 5 μl reactions. We found that the false negative rate decreased significantly for samples containing 10 and 5 copies, with false negative rates ranging from 0.0% to 0.1%.

We also wanted to determine the utility of Ct-values for delineating true detection in low concentration samples from noise. The means and medians of the Ct-values from amplified wells in the LOD experiments are shown in Additional file 1: Table S3. The medians of the 10 copies and 5 copies samples in 10 μl reaction were statistically lower than water-only or human-only samples. However, the 1.8 copy samples did not have a median value that could be discriminated from the negative control distributions in either reaction volume, despite the approximately one cycle earlier amplifications observed for 5 and 10 copies in 5 μl reactions. Given these results, and the distribution of the Ct-values from each condition tested, we determined the Ct-values for ≥ 5 copies template (Additional file 8: Figure S4). Based on this, we further determined that a one standard deviation cutoff could be used to remove outlying values from a set of triplicate test result. The Ct-value distribution also supports an averaging approach of non-outlying quantified values to determine the best estimate of the true Ct-value using the FungiQuant triplicates in analysis.

## Discussion

In the current manuscript, we present our design and validation of FungiQuant, a broad-coverage TaqMan^®^ qPCR assay for quantifying total fungal load and reproducibly detecting 5 copies of the fungal 18S rRNA gene using triplicate 10 μl reactions. The *in silico* analysis was an important component of our validation of FungiQuant against diverse fungal sequence types, even though sequence matching is not a perfect predictor of laboratory performance [38]. Many factors are known to affect reaction efficiency, such as oligonucleotide thermodynamics, the type of PCR master mix used, and the template DNA extraction method. Thus, given the range of FungiQuant reaction efficiency against different fungal species, we expect FungiQuant to be more accurate in longitudinal than cross-sectional studies. Background nontarget genomic DNA is another factor known to affect assays targeting the conserved rRNA gene [39]. To address this, we have developed FungiQuant analysis guideline for differentiating random noise from true detection. Lastly, to address the potential presence of exogenous fungal DNA, we recommend the use of negative controls at each sample processing and analysis step.

With respect to FungiQuant LOD, it is worth noting that a concentration of 1.8 copies/μl of 18S rRNA gene is the equivalent of 0.5 fg/μl of *C. albicans* DNA, with the assumption of 55 18S rRNA gene copy number per haploid genome [40]. This concentration, using the published haploid genome size of 15.185 × 10^-3^ pg for *C. albicans* shows that 0.5 fg is the equivalent of 1/30 of a single *C. albicans* genome [40]. Using the same estimates, the 5-copy LOD of FungiQuant is thus the equivalent of 1.38 fg/μl of *C. albicans* DNA, or the 1/11 of a single *C. albicans* genome. Similar conversions of DNA concentration and genomic equivalents for LOD estimation for other fungal species can be performed accordingly; this can help to facilitate estimation of DNA concentrations and genomic equivalents of fungi present at levels below other quantitation approaches, including spectrometric and fluorimetric methods.

Use of a probe-based reporting mechanism is an important feature in FungiQuant in two respects. First, it enhances the quantitative capability of FungiQuant, and secondly, improves assay specificity. An example illustrating the advantage of probe-based reporting is the comparison of FungiQuant with an intercalating dye-based qPCR assay, which had amplification efficiencies ranging from 67-103% and a LOD of 500pg of fungal DNA [30]. Additionally, the intercalating dye can generate amplification signal irrespective of amplicon size or composition.

In summary, we have developed and evaluated a new broad-coverage qPCR assay—FungiQuant—for diverse fungal detection and quantification that showed broad assay coverage and favorable quantitative parameters. A limitation of the current manuscript is the conversion from 18S rRNA gene copy number to the number of cells or biomass. In order to generate an estimated genomic equivalent, improved knowledge of 18S rRNA gene copy number of diverse fungi is required. And given that 18S rRNA gene copy number varies among fungal species and even among strains or over the lifetime of the fungi [41-43], this challenge will likely to persist. In addition to the design and validation of a broad-coverage fungal qPCR assay, our manuscript also sought to address basic limitations of evaluating combined primer and probe coverage, as well as generating reference standards for absolute quantification. Our approach of evaluating assay coverage by considering the primer and probe sequences as a single unit is appropriate and necessary. Additionally, our approach of quantifying plasmid standards using the intrinsic property of real-time PCR is another important step for any absolute quantification experiments using qPCR.

## Competing interests

The authors have declared that no competing interests exist.

## Authors’ contributions

CML contributed to the overall study design, the acquisition, analysis, and interpretation of data, and drafting the manuscript, SK participated in the bioinformatics analysis and assay design, AGA contributed to the analysis and interpretation of data; MGD and MA both contributed to the bioinformatics portion of the analysis, PRH, YTH, JDB, LJL, and CAG contributed to the acquisition and interpretation of laboratory data, PK conceived of the study and contributed to the overall study design, LBP contributed to the overall study design. All authors read and approved the final manuscript.

## Supplementary Material

Additional file 1 **Supplemental Methodological Details, Figure Legends, and Tables.** This supplemental file contains supplementary bioinformatics and laboratory details, figure legends for Figure S1, S2A-D, S3, and S4, and Tables S1-3. Click here for file

Additional file 2: Figure S1 Results of the *in silico* FungiQuant coverage analysis using the stringent criteria. Click here for file

Additional file 3: Table S4 Detailed results for FungiQuant using the stringent criteria. Click here for file

Additional file 4: Table S5 Detailed results for FungiQuant using the relaxed criteria. Click here for file

Additional file 5: Table S6 Detailed results for fungal species with perfect matches to *C. albicans* in the FungiQuant primer and probe region. Click here for file

Additional File 6: Figure S2A-C Coefficient of variance (CoV) distribution across FungiQuant assay dynamic range for mixed templates. Click here for file

Additional File 7: Figure S3A-D FungiQuant Standard curve amplification plots using additional types of templates. Click here for file

Additional File 8: Figure S4 The Ct-value distribution from 96-replicates for each low-copy target and negative control condition tested. Click here for file
