# Supplementary material for: FungiQuant: A broad-coverage fungal quantitative real-time PCR assay
Source: BMC Microbiol. 2012 Nov 8;12:255. doi: 10.1186/1471-2180-12-255 (PMC3565980; doi:10.1186/1471-2180-12-255)
Supplement: Additional file 1 — Supplemental Methodological Details, Figure Legends, and Tables. This supplemental file contains supplementary bioinformatics and laboratory details, figure legends for Figure S1, S2A-D, S3, and S4, and Tables S1-3. [file 1471-2180-12-255-S1.doc]

**Supplemental Methodological Details, Figure Legends, and Tables.**

**Supplementary Methodological Details**

**A. Numerical coverage analysis.** We assessed the proportion of unique operational taxonomic units (OTUs) that are perfect sequence matches to FungiQuant. We assessed the FungiQuant numerical coverage as follows: we identified the Assay Perfect Match sequence IDs using the select matching condition (i.e., the stringent or the relaxed criterion), where an Assay Perfect Match = {*Forward Primer Perfect Match*} ∩ {*Reverse Primer Perfect Match*} ∩ {*Probe Perfect Match*}. We binned and extracted the taxonomic information for each Assay Perfect Match using its correlated Genbank accession numbers. We began by generating the numerator by tabulating all species that had at least Assay Perfect Match and the denominator by summing total number of unique species in our eligible sequence set. We reiterated this numerical coverage analysis for subsequent taxonomic levels until the phylum level analysis was completed.

**B. Taxonomic coverage analysis.** We generated a comprehensive list of unique OTUs that are perfect sequence matches to FungiQuant. We assessed the FungiQuant taxonomic coverage as follows: first, we subtracted the Assay Perfect Match sequence IDs from all eligible sequences to produce the Assay Non-Perfect Match sequence IDs. Next, we concatenated taxonomic information of all eligible sequences and dereplicated all Assay Perfect Match and Assay Non-Perfect Match sequence IDs to generate the final output. This was repeated at subsequent taxonomic levels until the phylum level analysis was completed. The full taxonomic coverage results were not presented in the manuscript due to its extensive size but are in Supplemental Files 1-2.

**C.** **Generation of normalized 18S rRNA gene plasmid standards.** We generated the 18S rRNA gene amplicon using *C. albicans* genomic DNA as the template, with the forward (5’-GGAGARRGAGCCTGAGA-3’) and reverse (5’-CTAGGNATTC-CTCGTTSAAG-3’) primers. We visualized the amplicon using SYBR 2% agarose gel. We used the resultant PCR amplicons as the target gene insert using the pCR®2.1 TOPO® vector (Invitrogen Corp., Carlsbad, CA, USA), which we purified using the QIAprep Spin Miniprep Kit (Qiagen Inc., Valencia, CA, USA). We verified insert sequence using capillary electrophoresis using on the 3130 Genetic Analyzer platform (Applied Biosystems, Carlsbad, CA, USA). Next, we quantified the plasmid standards using a qPCR assay that targets a conserved region of the plasmid on the LightCyler® 480 Real-Time PCR System (Roche Applied Science, Indianapolis, IN, USA). Based on the resultant Cp-value, we normalized the plasmid standards using the dilution factor 2^ΔCp^, where ΔCp = 10 – (Cp value of non-normalized cloned plasmids).

**D. Diverse fungal DNA used in FungiQuant laboratory evalution**. Fungal DNA was obtained from the American Type Culture Collection for *Debaryomyces hansenii* ATCC 36239, *Lodderomyces elongisporus* ATCC 11503, and *Schizosaccharomyces pombe* 26189. Additionally, DNA from clinical isolates of *Absidia corymbifera, Acremonium strictum*, *Aspergillus flavus*, *Aspergillus fumigatus*, *Aspergillus niger*, *Aspergillus versicolor, Aureobasidium pullulans, Candida famata, Candida guilliermondii, Candida haemulonii, Candida intermedia, Candida quercitrusa, Candida tropicalis, Chaetomium globosum*, *Cunninghamella bertholletiae, Elaphomyces decipiens*, *Exophiala dermatitidis*, *Fusarium equiseti*, *Fusarium oxysporum*, *Fusarium solani*, *Geotrichum candidum, Microsporum canis*, *Neurospora crassa*, *Paecilomyces lilacinus*, *Paecilomyces sinensis*, *Paecilomyces variotii*, *Penicillium marneffei, Pichia ohmeri, Rhizopus microspores, Rhizopus oryza, Saccharomycopsis crataegensis, Scedosporium apiospermum, Sporothrix schenckii*, *Stephanoascus ciferrii, Trichophyton mentagrophytes*, *Trichophyton rubrum* were kindly provided by National Taiwan University, Taipei, Taiwan. Verification of clinical isolates was accomplished by sequencing of the internal transcribed spacer region (ITS), the intergenic spacer region (IGS), or a combination of ITS and the 18S rRNA gene.

Additional DNA from environmental isolates of *Agaricus spp., Alternaria spp., Cladosporium cladosporioides*, *Clavulina coralloides*, *Coprinus spp.* *Cortinarius spp.*, *Cytospora chrysosperma, Endoconidioma spp., Geopora spp., Hebeloma crustuliniforme group, Melanogaster spp.,* *Phoma herbarum*, *Pleurotus ostreatus*, *Rhizopogon spp.*, *Rhodotorula mucilaginosa*, *Rhodotorula slooffiae*, *Sclerogaster xerophilus*, *Sedecula pulvinata*, *Tricholoma populinum*, *Trichosporon asahii, Trichosporon asteroids, Trichosporon cutaneum, Trichosporon dermatis, Trichosporon faecale, Trichosporon montevideense, Trichosporon mucoides, Trichosporon ovoides, Xanthomendoza galericulata,* and *Lactarius spp.* were kindly provided by Northern Arizona University, Flagstaff, Arizona. Verification of environmental isolates was performed based on morphology and/or ITS sequence verification.

**FungiQuant laboratory quantitative validation data analysis.** Using the data generated, the following assay parameters were calculated: 1) inter-run assay coefficient of variation (CoV) for copy number and Ct-value, 2) average intra-run assay CoV for copy number and Ct. value, 3) assay dynamic range, 4) average reaction efficiency, and 5) correlation coefficient (*r^2^*-value). The limit of detection was not defined for the pure plasmid standards experiments due to variability in reagent contamination. At each plasmid standard concentration, the Ct standard deviation across all standard curves over three runs was divided by the mean Ct-value across all standard curves over three runs to obtain the inter-run assay CoV. The CoV from each standard curve from each run (i.e., nine CoV were used in the calculation for each condition tested) were used to calculate the average and the standard deviation of the intra-run CoV. Linear regression of each standard curve across the full dynamic range was performed to obtain the slope and correlation coefficient values. The slope was used to calculate the reaction efficiency using Efficiency = 10^(-1/slope)^-1. Of note, for each triplicate reaction with Ct standard deviation >0.2, the triplicates were compared and if a clear outlier was present (ΔCt > 0.2 from other two replicates), the outlier well was excluded from analysis. Amplification profiles of pure and mixed templates tested were annotated and presented in Fig. 2A-B and Supplementary Fig. 2A-D. Results from laboratory quantitative validation using all conditions tested were summarized in Table 4. Detailed results of inter- and intra-run coefficient of variation for Ct-value and copy number were presented for all conditions tested in Fig. 3 and Supplementary Fig. 5A-C using scatterplots generated with the *vegan* package in R

**Fig. S1.** **Results from *in silico* coverage analysis of the FungiQuant assay using the stringent criterion against 993 genera and 9 phyla showing broad coverage.** On the circular 18S rRNA gene-based maximum parsimony phylogeny, each of the covered and uncovered phyla by the FungiQuant assay, based on the stringent criteria, is annotated with the genus-level numerical coverage in parenthesis below the phylum name. Each genus-level numerical coverage annotation consists of a numerator (i.e., the number of covered genus for the phylum), a denominator (i.e., the total number of genera eligible for sequence matching for the phylum), and a percentage calculated using the numerator and denominator values.

**Fig. S2A-D.** **Standard curve amplification profiles of the FungiQuant against mixed templates in seven ten-fold dilutions in 10 µl reactions.** The overall amplification profiles are not significantly different between the different reaction volumes over the assay dynamic range of 25 copies to 10^7^ copies of 18S rRNA gene, even in the presence of varying amounts of human DNA at up to 10 ng.

**Fig. S3A-C.** **Inter- and intra-run coefficient of variation (CoV) for 10 µl and 5 µl reactions using seven ten-fold dilutions and normalized plasmid standards at 10^9^ copies/µl calculated using data from multiple runs.** The data is presented for both copy number (*solid line*) and Ct-value (*dashed line*). As would be expected, the CoV is higher for copy number than for Ct-value and is also higher for inter-run than for intra-run.

**Fig. S4. Ct-value distributions of FungiQuant tested against low copy templates in 96-replicates each of 10 µl and of 5 µl reactions.** The experimental Ct-values of 5 copies (in purple) and 10 copies of 18S rRNA gene could be distinguished from 1.8 copies (in green) and 10 ng of human DNA (in blue). The ≥ 5 copies templates also demonstrated near-normal distribution as shown.

**Table S1.** The frequency of positive amplifications for each experimental condition based on 96 replicates. The sensitivity and specificity for the positive and negative controls is shown.

| **Experimental Conditions** | **No. of Positive Amplifications** | **Ct-value** |
| --- | --- | --- |
| 10 μl Reaction |  |  |
| 10 copies | 0 | 96 (Sensitivity = 100%) |
| 5 copies | 0 | 96 (Sensitivity = 100%) |
| 1.8 copies | 68 | 28 (Sensitivity = 29%) |
| Human 10 ng | 87 (Specificity = 91%) | 9 |
| No Template | 95 (Specificity = 99%) | 1 |
| 5 μl Reaction |  |  |
| 10 copies | 2 | 94 (Sensitivity = 98%) |
| 5 copies | 2 | 94 (Sensitivity = 98%) |
| 1.8 copies | 74 | 22 (Sensitivity = 23%) |
| Human 10 ng | 79 (Specificity = 82%) | 17 |
| No Template | 89 (Specificity = 93%) | 7 |

**Table S2.** Results of the median (IQR) and mean (SD) of Ct-values from each experimental condition.

| **Experimental Conditions** | **No. of Positive Amplifications** | **Ct-value**  **Median (IQR)** | **Ct-value**  **Mean (SD)** |
| --- | --- | --- | --- |
| 10 μl Reaction |  |  |  |
| 10 copies | 96/96 | 34.1 (33.8, 34.3) | 34.12 (0.50) |
| 5 copies | 96/96 | 36.5 (35.9, 37.1) | 36.70 (1.40) |
| 1.8 copies | 28/96 | 38.3 (38.0, 39.1) | 39.06 (2.38) |
| Human 10 ng | 9/96 | 38.3 (37.7, 39.1) | 38.83 (1.62) |
| Human 50 ng | 13/48 | 38.6 (38.0, 39.6) | 39.16 (1.80) |
| Human 150 ng | 27/48 | 38.4 (38.1, 39.1) | 38.40 (1.28) |
| No Template | 1/96 | 38.7 (38.7, 38.7) | 38.72 (---) |
| 5 μl Reaction |  |  |  |
| 10 copies | 94/96 | 32.9 (32.4, 33.5) | 33.20 (1.49) |
| 5 copies | 94/96 | 34.8 (34.1, 35.9) | 35.33 (1.97) |
| 1.8 copies | 22/96 | 37.9 (36.9, 39.1) | 38.72 (3.07) |
| Human 10 ng | 17/96 | 37.0 (36.3, 37.7) | 36.43 (2.88) |
| Human 150 ng | 33/48 | 36.5 (36.2, 37.9) | 36.66 (1.39) |
| No Template | 6/96 | 37.5 (32.9, 37.9) | 36.07 (3.29) |

**Table S3. Comparison of human 18S rRNA gene sequence with FungiQuant primer and probe sequences.**

|  |  |  |
| --- | --- | --- |
| Human | ggaaacctcacccggcccgg | |
| FungiQuant-F | GGRAAACTCACCAGGTCCAG | |
|  |  |  |
| Human | Ccccgatccccatcacga | |
| FungiQuant-R | GSWCTATCCCCAKCACGA | |
|  |  |  |
| Human | tggtgcatggccgtt | |
| FungiQuant-prb | TGGTGCATGGCCGTT | |

**References**

1. Oksanen J, Blanchet F, Kindt R, Legendre P, O'Hara R, GL. S, Solymos PM, Stevens H, Wagner H: **vegan: Community Ecology Package. R package version 1.17-8.** In*.*; 2011.

2. R Development Core Team: **R: A language and environment for statistical computing**. Vienna: R Foundation for Statistical Computing; 2008.
