# Supplementary figures and images for: FungiQuant: A broad-coverage fungal quantitative real-time PCR assay
Source: BMC Microbiol. 2012 Nov 8;12:255. doi: 10.1186/1471-2180-12-255 (PMC3565980; doi:10.1186/1471-2180-12-255)

Basidiomycota: Class Dacrymycetes  
100% (5/5)  
Basidiomycota: Class Tremellomycetes  
90.91% (30/33)

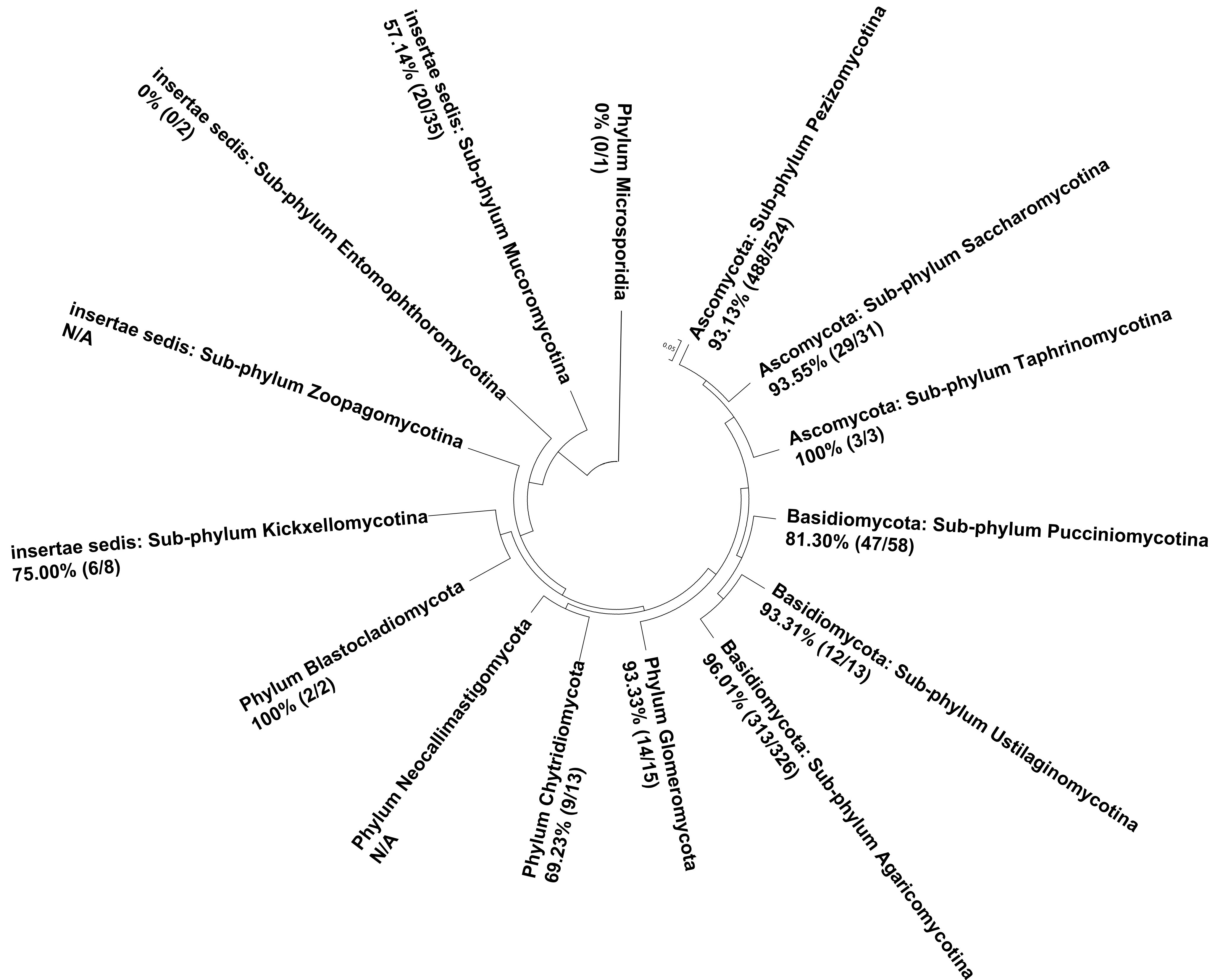

Supplement: Additional file 2: Figure S1 — Results of the in silico FungiQuant coverage analysis using the stringent criteria. [file 1471-2180-12-255-S2.pdf]

Assay Coefficient of Variance

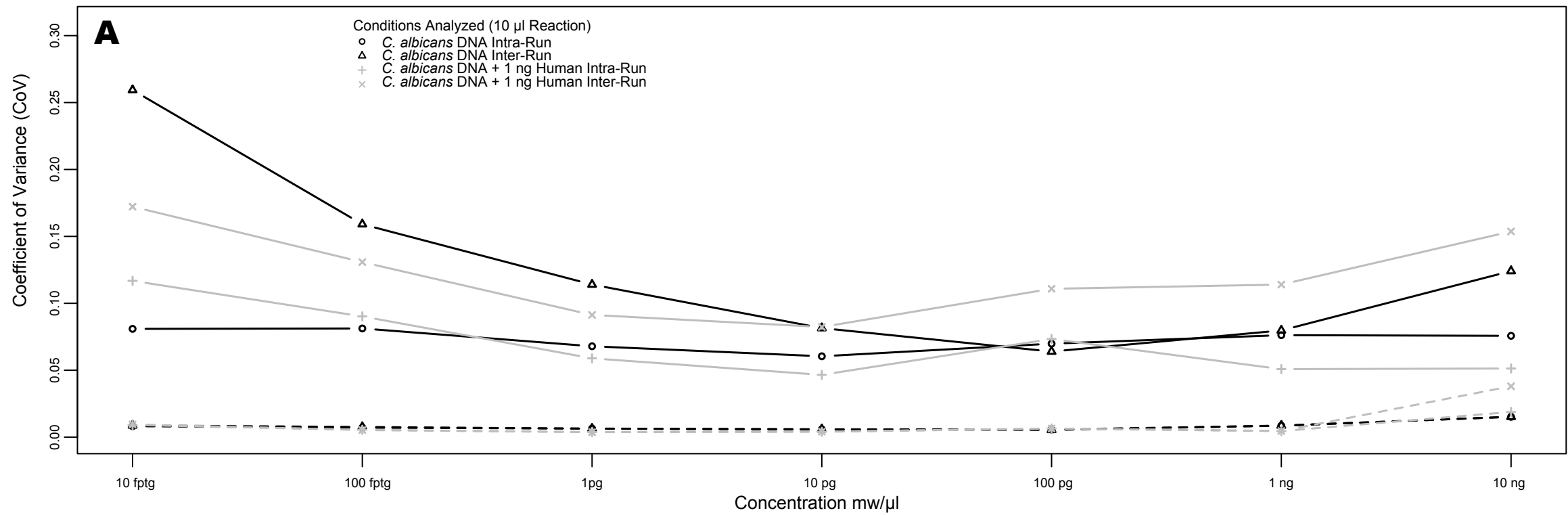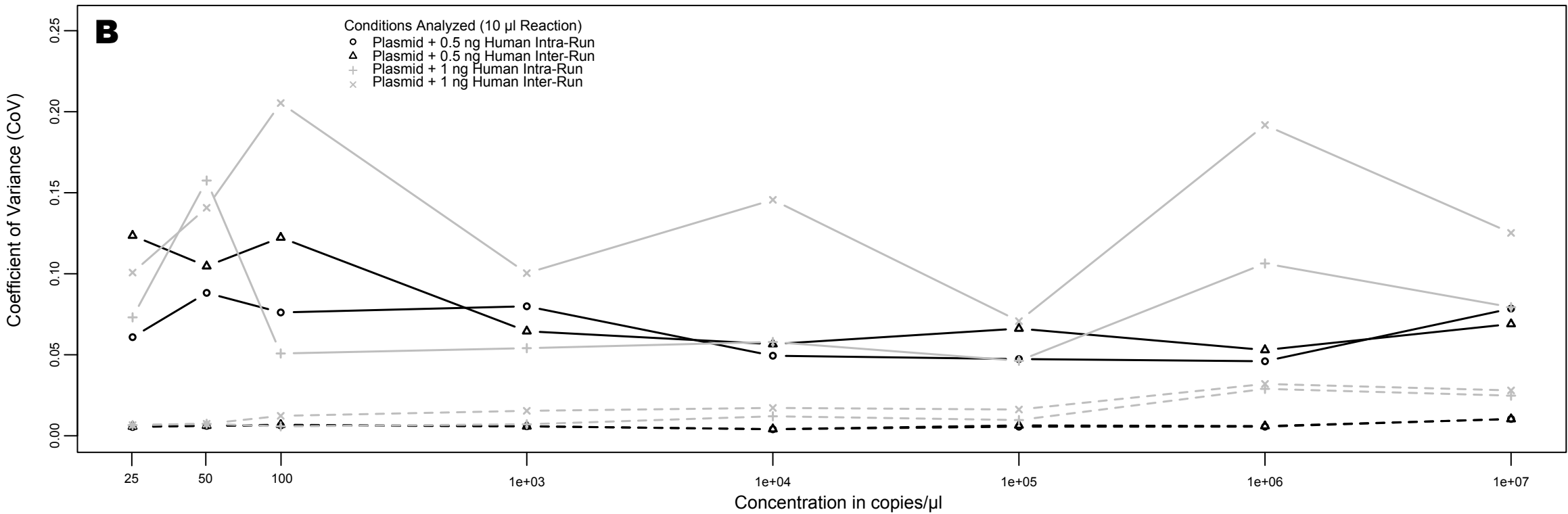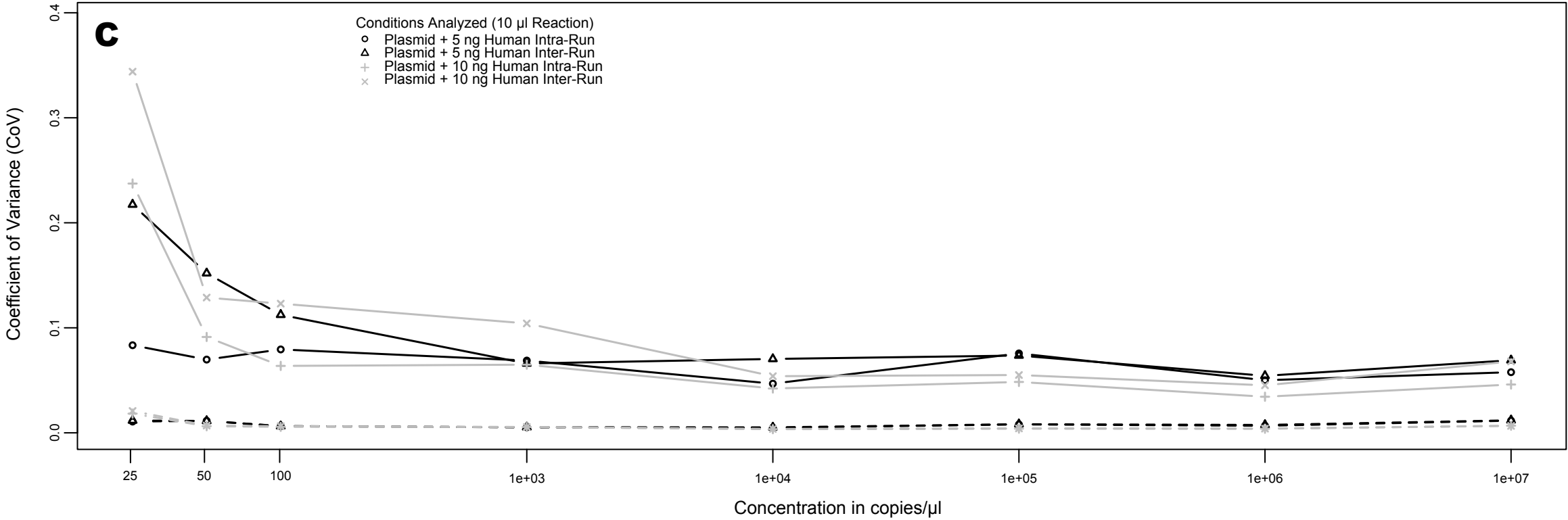

Supplement: Additional File 6: Figure S2A-C — Coefficient of variance (CoV) distribution across FungiQuant assay dynamic range for mixed templates. [file 1471-2180-12-255-S6.pdf]

**A. 10  $\mu$ l Reaction**

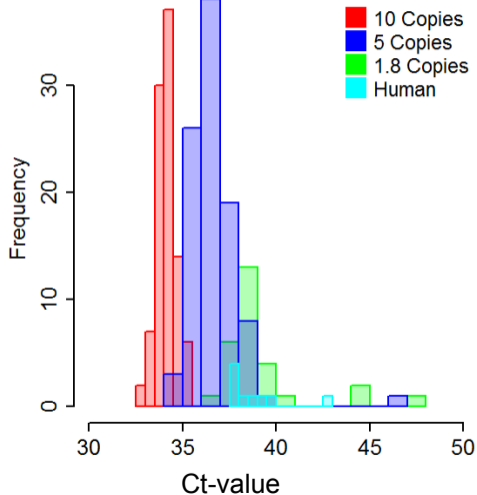

**B. 5  $\mu$ l Reaction**

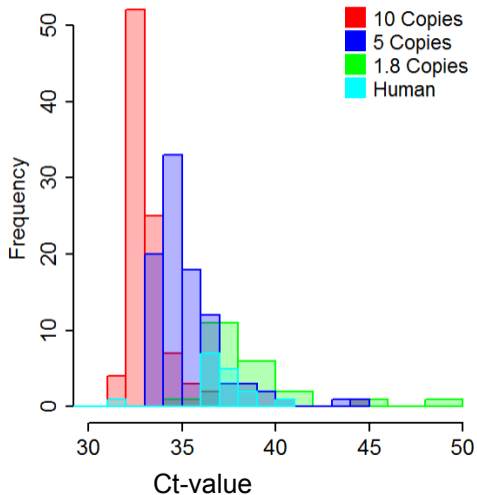

Supplement: Additional File 8: Figure S4 — The Ct-value distribution from 96-replicates for each low-copy target and negative control condition tested. [file 1471-2180-12-255-S8.pdf]
