# Supplementary material for: FungiQuant: A broad-coverage fungal quantitative real-time PCR assay
Source: BMC Microbiol. 2012 Nov 8;12:255. doi: 10.1186/1471-2180-12-255 (PMC3565980; doi:10.1186/1471-2180-12-255)
Supplement: Additional File 7: Figure S3A-D — FungiQuant Standard curve amplification plots using additional types of templates. [file 1471-2180-12-255-S7.pdf]

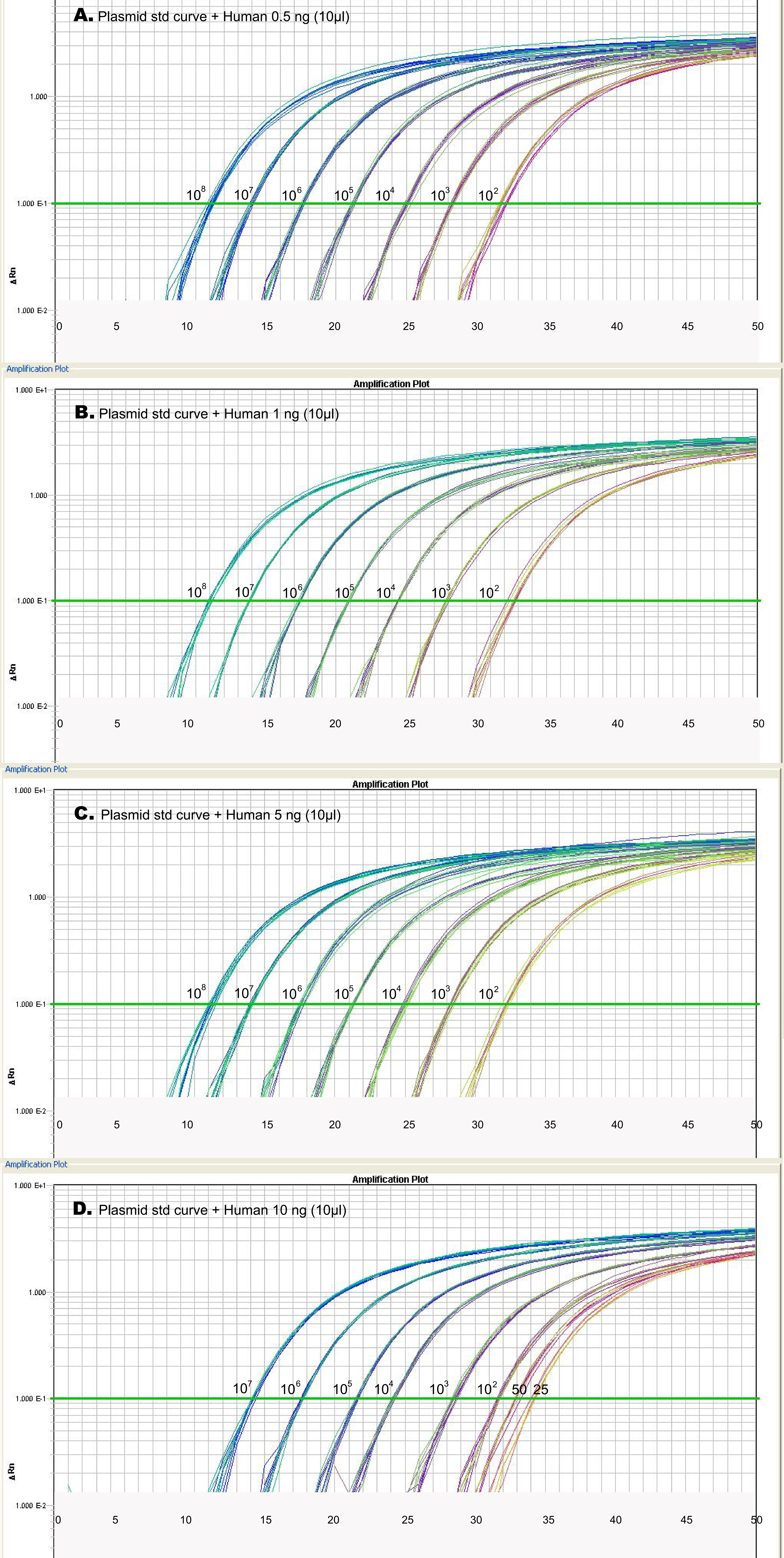

A. Plasmid std curve + Human 0.5 ng (10µl)
Amplification Plot
Amplification Plot
B. Plasmid std curve + Human 1 ng (10µl)
Amplification Plot
Amplification Plot
C. Plasmid std curve + Human 5 ng (10µl)
Amplification Plot
Amplification Plot
D. Plasmid std curve + Human 10 ng (10µl)
ΔRn
1.000 E+1
1.000
1.000 E-1
1.000 E-2
10^8 10^7 10^6 10^5 10^4 10^3 10^2
10^7 10^6 10^5 10^4 10^3 10^2 50 25
0 5 10 15 20 25 30 35 40 45 50
